# Dynamics of the interaction between the receptor-binding domain of SARS-CoV-2 Omicron (B.1.1.529) variant and human angiotensin-converting enzyme 2

**DOI:** 10.7717/peerj.13680

**Published:** 2022-07-05

**Authors:** Priya Antony, Amie Jobe, Ranjit Vijayan

**Affiliations:** 1 Department of Biology, College of Science, United Arab Emirates University, Al Ain, Abu Dhabi, United Arab Emirates; 2 The Big Data Analytics Center, United Arab Emirates University, Al Ain, Abu Dhabi, United Arab Emirates; 3 Zayed Center for Health Sciences, United Arab Emirates University, Al Ain, Abu Dhabi, United Arab Emirates

**Keywords:** SARS-CoV-2, Omicron, B.1.1.529, Spike protein, Angiotensin-converting enzyme 2, Molecular dynamics

## Abstract

**Background:**

The COVID-19 pandemic is still a global public health issue. Omicron, a SARS-CoV-2 B.1.1.529 variant, has raised concerns about transmission and vaccine effectiveness. Omicron currently has the greatest number of variantions.

**Methods:**

To gain a better understanding of the significance of these variations and the dynamics of the interaction between the Omicron spike (S) protein and its human host factor angiotensin-converting enzyme 2 (ACE2), triplicate 500 ns molecular dynamics simulations were run using the structure of the S protein’s receptor-binding domain (RBD) in complex with ACE2. The interaction and binding energy, determined using the molecular mechanics—generalized Born surface area approach, were compared to the original SARS-CoV-2 and the B.1.617 variant.

**Results:**

Though mutations K417N and G496S in the S protein RBD disrupt interactions found in the original SARS-CoV-2 complex, mutations Q493R and N501Y introduce interactions not found in the original complex. Interaction at a key viral hotspot and hydrophobic contacts at ACE2’s N-terminus were preserved, but intermolecular hydrogen bonds and polar contacts in the S-ACE2 interface were lower than in the original SARS-CoV-2 interface.

## Introduction

The ongoing coronavirus disease 2019 (COVID-19) pandemic, caused by the severe acute respiratory syndrome coronavirus 2 (SARS-CoV-2) coronavirus, remains a major global health concern. Since its emergence in late 2019, the virus has spread globally; the total number of diagnosed cases now exceeds 530 million and the number of deaths is close to the six million mark (https://coronavirus.jhu.edu, accessed on 5 June 2022).

Rapid development and delivery of a variety of vaccines have had a significant impact on limiting the pandemic’s severity to a large extent. The emergence of new SARS-CoV-2 mutant strains with improved infectivity and transmission capacity, on the other hand, raises significant challenges in fully containing the virus (Harvey et al., 2021).

Like other viruses, SARS-CoV-2 mutates and evolves as it replicates. As there is no mismatch repair mechanism, the mutation rate is extremely high (Domingo et al., 2021). However, only a small percentage of these mutations are expected to change the virus’s functional properties, resulting in improved infectivity, disease severity, transmissibility, and interaction with host immunity. Several viral variants, harboring various sets of mutations have been reported (Leung et al., 2021; Sabino et al., 2021; Tegally et al., 2021).

Mutations that have increased the virus’s contagiousness have persisted. For example, the D614G mutation in the SARS-CoV-2 spike (S) protein, which was one of the first viral variants discovered, now predominates globally (Korber et al., 2020). The recombination process has also resulted in a variety of SARS-CoV-2 variants with high transmissibility and immune escape (Zhou & Wang, 2021).

In early November 2021, a new viral variant was reported from genomic sequencing of samples collected from Botswana and South Africa (Karim & Karim, 2021). In comparison to the original SARS-CoV-2, the new variant belongs to the Pango lineage B.1.1.529 and has a large number of mutations in the S region. The World Health Organization (WHO) designated this variant as a variant of concern on November 26, 2021, and named it “Omicron” (WHO). Omicron is one of the most heavily mutated variants characterized by 30 missense mutations, three small deletions, and one small insertion in the S protein (A67V, Δ69–70, T95I, G142D, Δ143–145, Δ211, L212I, ins214EPE, G339D, S371L, S373P, S375F, K417N, N440K, G446S, S477N, T478K, E484A, Q493R, G496S, Q498R, N501Y, Y505H, T547K, D614G, H655Y, N679K, P681H, N764K, D796Y, N856K, Q954H, N969K, and L981F).

**Figure 1:**
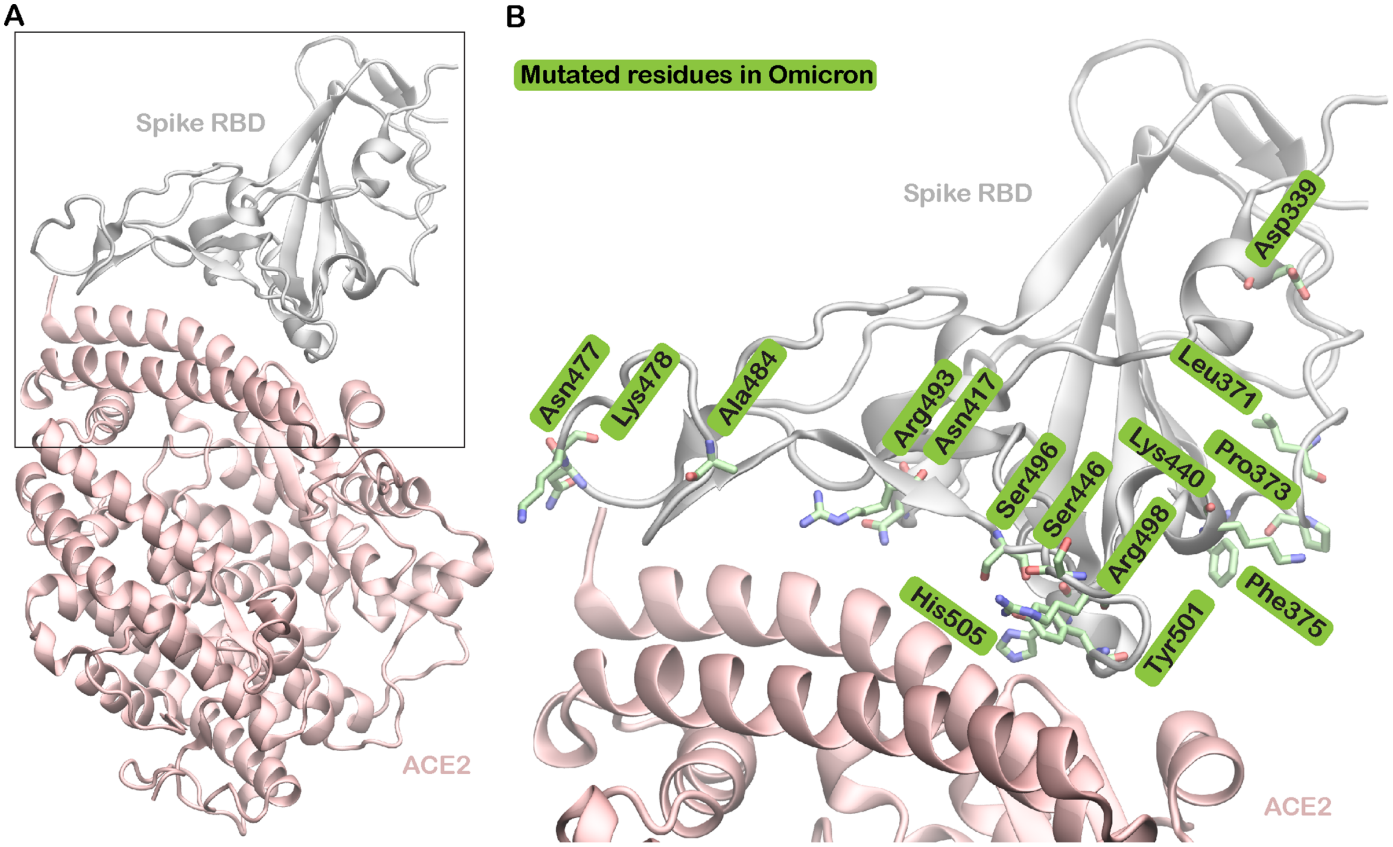
Omicron spike protein receptor binding domain bound to angiotensin-converting enzyme 2 (ACE2). The protein chains are shown in cartoon representation. (A) Structure of the Omicron spike (S) protein (gray) ACE2 bound (pink). (B) The boxed region in A has been enlarged to show the mutations found in the Omicron variant.

As the binding of the S protein receptor-binding domain (RBD) to the human host receptor angiotensin-converting enzyme 2 (ACE2) is considered the most important step in viral infection, any changes in this region, particularly within the receptor-binding motif (RBM), may result in improved or weakened virus binding to the host (Lan et al., 2020). Furthermore, as the S protein was the primary choice for the design of vaccines, these mutations could also affect vaccine efficacy. In the Omicron variant, 15 mutations appear in the RBD (S protein residues 319–541) with 10 of these in the RBM (residues 438–506) (Fig. 1). Notably, some of these mutations, including S477N, Q498R, and N501Y, have previously been linked to increased transmissibility, infectivity, and immune escape (Greaney et al., 2021; Huang et al., 2021; Singh et al., 2021). Several reports have now demonstrated Omicron’s immune escape capability (Zhang et al., 2021; Dejnirattisai et al., 2022; Hu et al., 2022). This could be attributed to mutations in the RBD region and the N terminal domain, highlighting the urgent need for more potent vaccines and antibodies (Andreano et al., 2021).

A deeper understanding of the interactions between the Omicron variant and ACE2 could shed light on its binding efficiency and transmissibility. This is critical for determining the efficacy of currently available vaccines and, if necessary, designing new vaccines to combat this and other emerging variants. Molecular dynamics (MD) simulations have become an indispensable tool for understanding the structural and molecular mechanisms of SARS-CoV-2 infection in recent years (Padhi, Rath & Tripathi, 2021). Our previous MD studies have evaluated the dynamics of the interactions between the S protein of the original SARS-CoV-2 and the B.1.617 variant with ACE2 (Ali & Vijayan, 2020; Antony & Vijayan, 2021). Using multiple 500 ns MD simulations and binding free energy calculations, we investigate the structural stability, binding affinity, and intermolecular polar and hydrophobic contacts between the RBD of the Omicron variant and human ACE2.

## Materials and Methods

Coordinates of the three-dimensional X-ray crystal structures of the SARS-CoV-2 RBD (S-RBD) in complex with human ACE2 were retrieved from the Protein Data Bank (PDB; PDB ID: 7WBP; Han et al., 2022). The structure was pre-processed with the Schrödinger Protein Preparation Wizard (Schrödinger, LLC, New York, NY, USA). The protein preparation step included assigning proper bond orders, adding disulfide bonds, adjusting ionization states, correcting disoriented groups, capping termini, adding sidechains and missing atoms, assigning partial charges, and removing unwanted metals, cofactors, and water molecules. Additionally, hydrogen atoms were added, and a standard protonation state at pH 7 was used. Using the default settings, hydrogen bonds were optimized and the structure’s energy was minimized. The Omicron S-RBD in complex with ACE2 was placed in an orthorhombic box of dimension 125 × 125 × 125 Å and solvated with single point charge water molecules using the Desmond System Builder (Schrödinger, LLC, New York, NY, USA). The simulation setup was neutralized with counterions in each case, and a salt concentration of 0.15 M NaCl was maintained. Desmond (Bowers et al., 2006) was used to run 500 ns MD simulations of the complex after assigning initial velocities to each atom based on different random seeds. The simulation systems were described using the OPLS forcefield. Before the production run, all simulation systems underwent Desmond’s default eight-stage relaxation protocol. The Nosé–Hoover thermostat and the isotropic Martyna–Tobias–Klein barostat were used to keep the temperature at 300 K and the pressure at 1 atm constant, respectively (Martyna, Klein & Tuckerman, 1992; Martyna, Tobias & Klein, 1994). The smooth particle mesh Ewald approach was used to evaluate long-range Coulombic interactions, and the short-range cut-off was set at 9.0 Å. (Essmann et al., 1995). A time-reversible reference system propagator algorithm (RESPA) integrator was used with an inner time step of 2.0 fs and an outer time step of 6.0 fs (Tuckerman, Berne & Martyna, 1992). The binding free energy of the mutant complex was computed based on the molecular mechanics—generalized Born surface area (MM-GBSA) approach by extracting frames at 5 ns intervals from MD simulation trajectories. The MM-GBSA-based binding free energy was then calculated using Schrödinger Prime and the VSGB 2.0 solvation model (Li et al., 2011). Packaged and custom scripts were used to analyze simulation data. Visual Molecular Dynamics version 1.9.3 (Humphrey, Dalke & Schulten, 1996) was used to generate structural images, and the R version 3.6.3 (https://www.r-project.org, accessed on 8 July 2020) was used to plot graphs.

## Results and Discussion

**Figure 2:**
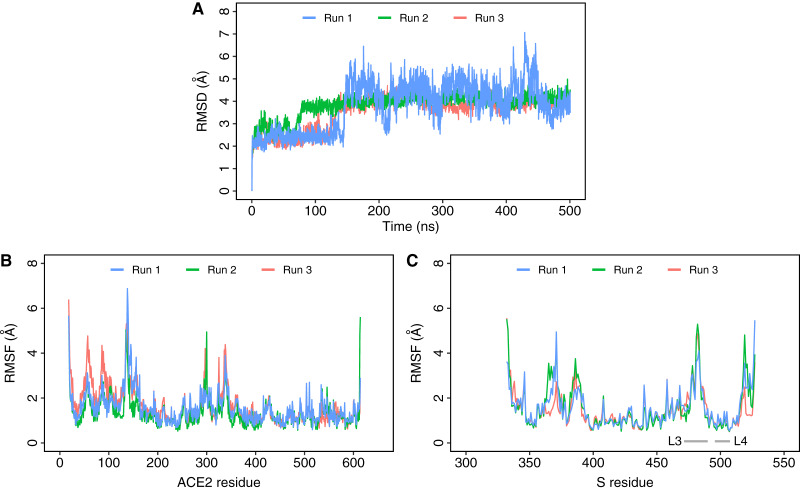
Protein root mean square deviation (RMSD) and root mean square fluctuation (RMSF). (A) RMSD of backbone Ca atoms of Omicron S protein RBD bound to ACE2 from three independent 500 ns simulations of the complex. Figure S1 shows the RMSD of ACE2 and S separately. (B) RMSF of Cα atoms of ACE2 residues. (C) RMSF of Ca atoms of Omicron S-RBD residues. Loops 3 (L3; residues 471–491) and 4 (L4; residues 495–508) are shown and marked with a gray bar.

To get a deeper insight into the dynamics of the interaction between the Omicron variant and ACE2, triplicate 500 ns MD simulations were performed. This was compared to simulations of the original wild type complex (PDB ID: 6M0J) that we reported previously (Ali & Vijayan, 2020). The root mean square deviation (RMSD) of the complex increased slightly during the initial phase but quickly equilibrated after a few nanoseconds and remained below 5 Å in all three runs (Fig. 2A). A closer examination of the specific RMSDs of the ACE2 and S proteins (Fig. S1) and the MD trajectories revealed that the increase in RMSD in runs 2 and 3 were caused by a closing motion of ACE2’s active site (Trozzi et al., 2022), while the change in RMSD in run 1 was caused by a tilting of the spike protein (Fig. S2). The structural compactness of the complex was also maintained in the simulations similar to the wild type as evident from the radius of gyration (Rg; Fig. S3) and solvent-accessible surface area (SASA; Fig. S4). The drop in ACE2 SASA in runs 2 and 3 was due to the closing motion of the ACE2 active site. Residue level protein fluctuation was assessed using root mean square fluctuation (RMSF) (Figs. 2B and 2C). The binding interface of S-RBD consists of four loop regions—loop 1: residues 438–450, loop 2: residues 455–470, loop 3: residues 471–491, and loop 4: residues 495–508 (Williams et al., 2021). An examination of the RMSF plots (Fig. 2C) revealed that residues in the loop 3 and loop 4 regions fluctuated more than those in the wild type simulations (Ali & Vijayan, 2020). The fluctuation of the ACE2 backbone was comparable throughout the simulation period. Similar to the wild type, most of the fluctuations were observed in residues located in the loop 3 and loop 4 regions of the S structure (Fig. 2C). As this region is critical for binding to a host receptor, mutations could affect flexibility and, in turn, the ability to bind effectively to the host target protein.

**Figure 3:**
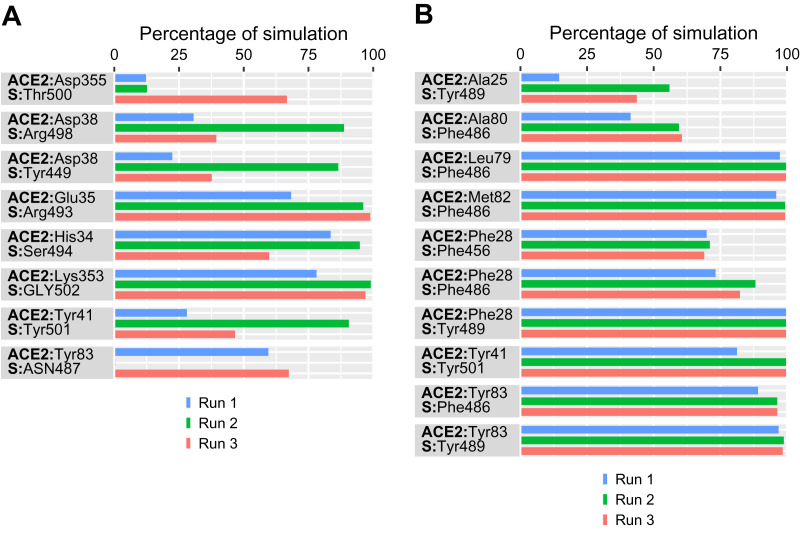
Percentage of simulation time intermolecular contacts were maintained between angiotensin-converting enzyme 2 (ACE2) and Omicron spike (S) protein residues from three independent 500 ns molecular dynamics simulations. (A) Polar contacts between ACE2 and S residues. (B) Hydrophobic contacts between ACE2 and S residues.

In simulations of the Omicron-protein complex, several intermolecular polar and hydrophobic interactions were observed to form, break, and reform. The data generated here was compared to our previously reported wild type data to assess the effect of Omicron variations on the molecular level interaction of SARS-CoV-2 RBD and ACE2 (Ali & Vijayan, 2020). Figure 3 summarizes the residue level interactions that were retained for at least 50% of the simulation time in at least one simulation. Notably, the mutations introduced in the Omicron variant disrupted the majority of the defining interactions of SARS-CoV-2 that conferred stable and more favorable binding to ACE2 compared to SARS-CoV. For example, the strong and stable salt bridge formed by Lys417 of S-RBD with Asp30 of ACE2 was disrupted by the K417N mutation (Fig. 3A). The Omicron Asn417 did not form any observable interaction with ACE2. This is consistent with a single K417N substitution being reported to reduce S-ACE2 interaction four-fold *via* lower binding and causing faster displacement (Laffeber et al., 2021). Similarly, the Phe456 and Tyr489 hydrophobic contacts with Thr27 of ACE2 in the wild type (Ali & Vijayan, 2020) were missing in the Omicron variant (Fig. 3B). Though Phe456 and Tyr489 are conserved in Omicron, the hydrophobic contact between S:Phe456 and ACE2:Thr27 in the wild type was replaced with interactions with ACE2:Phe28 in Omicron; and interaction of S:Tyr489 with ACE2:Thr27 in the wild type was replaced by contacts with Phe28 and Tyr83 of ACE2 in Omicron (Fig. 3B).

Among the three stable hydrogen bonds observed in the wild type between Tyr449, Gln493, and Gln498 of SARS-CoV-2 and Asp38, Glu35, and Lys353 of ACE2 (Ali & Vijayan, 2020), two interactions Tyr449 and Arg493 with Asp38 and Glu35 were found to be preserved in the Omicron variant following the Q493R substitution (Fig. 3A), with slightly higher contact time. As Q493 is in the antibody binding region, substitution to a positively charged residue, such as arginine or lysine, is likely to affect antibody binding (Focosi et al., 2021). Laurini et al. (2021) reported an interface stabilizing effect of the Q493K mutation, and immune evasion through monoclonal antibody neutralization was documented by Starr et al. (2021) (Laurini et al., 2021). Furthermore, the Q493K/R mutation was found to be highly resistant to monoclonal antibodies (mAbs; Lennerstrand & Palanisamy, 2021).

Looking at the effect of the mutations in the Omicron S-RBD on polar interactions, a key interaction preserved in both the wild type and Omicron was the interaction involving residue Gln493 of SARS-CoV-2 and Glu35 of ACE2. Interestingly, the Q493R RBM variant now results in a more stable salt bridge in Omicron with Glu35 of ACE2. The only other residue of the mutant that forms polar interactions with ACE2 is Tyr501 following the N501Y substitution in the RBM; this is through a π–π contact with Tyr41 of ACE2 with significant contact time in one simulation (Fig. 3A). When compared to the earlier Alpha and Beta variants, the Omicron variant shares the N501Y mutation, which favors higher binding affinity and immune evasion (Antony & Vijayan, 2021).

Through systematic experimental screening of potential S-RBD variations, it was demonstrated that changing N501 to F, W, V, or Y could increase S-RBD engagement with ACE2 and thus improve viral infectivity (Starr et al., 2020; Luan, Wang & Huynh, 2021). Additionally, the N501Y substitution could induce conformational alterations in residues within the S-RBD-ACE2 interface. This is consistent with the finding that N501Y causes a significant shift in electrostatic interactions, which increases S-RBD binding affinity to ACE2 (Ali, Kasry & Amin, 2021).

Following the K417N mutation in Omicron, Asn417 fails to form a polar contact with Asp30 and His34 of ACE2 (Ali & Vijayan, 2020). However, in all three runs, Ser494 made strong polar contact with His34 of ACE2. Similarly, due to the Y505H variation in Omicron, the interaction of Tyr505 in the RBD with Glu37 in the wild type is no longer possible (Fig. 3A). It has been suggested that the strong vaccine escape capability of the Omicron variant could be attributed to its K417N, E484A, and Y505H RBD mutations (Chen et al., 2021). Aside from Q493R, which forms sustained interactions with Glu35, and N501Y, which introduces new interactions in Omicron, other RBM mutations, such as G446S and G496S, disrupt polar interactions found in the wild type. Gln42-Gly446, Lys353-Gly496, and Lys353-Gly498 are notable interactions in the wild type that are absent in Omicron (Ali & Vijayan, 2020).

With the Q498R mutation, the Omicron variant still retains its interaction with Asp38, similar to the wild type. The Q498R mutation has been shown to increase ACE2 affinity and is frequently used to counteract antibodies. Mutational screening of single RBD mutations revealed that Q498R had a negative effect on protein stability and binding (Starr et al., 2020); however, binding affinity was nearly four-fold increased when combined with N501Y compared to N501Y alone. This epistatic phenomenon was further validated in a double-mutant by computing the interaction energy between the two residues (Zahradník et al., 2021).

The G446S variation is one of the most disruptive to the RBD; the other two are G446D and G446V (Verma & Subbarao, 2021). This highlights the relevance of Gly446 of loop 1 in offering flexibility to the RBM. The Gln42-Gly446 interaction in the wild type had a moderate contact time in a single simulation in our study, whereas this interaction was absent in Omicron. The effect of G496S is unknown, but Gly496 interacts with one of the two viral hotspots, Lys353 of ACE2 in the native complex (Ali & Vijayan, 2020), an interaction that is missing in the mutant complex.

In the wild type, the interaction of the RBD with the two viral hotspots within ACE2—Lys31 and Lys353—were mediated by the interactions Lys31-Gln493, Lys353-Gly496, Lys353-Gln498, and Lys353-Gly502. Only the Lys353-Gly502 association is preserved in Omicron, with a high contact time in three consecutive runs (Fig. 3A), whereas in the wild type, this association was observed with a high contact time in a single run. The hydrogen bond between Asp355 of ACE2 and Thr500 of S was another interaction conserved in both the wild type and Omicron, with significant contact time in one simulation (Fig. 3A).

In the vicinity of the RBD, an interaction between Asn487 of S and Tyr83 of ACE2 was noted in Omicron with significant contact in two runs (Fig. 3A). In Omicron simulations, no other interactions involving residues surrounding the wild type RBD were observed. These absent interactions include Tyr453 of S with His34 of ACE2 and Tyr489 of S with Gln24 and Thr27 of ACE2 (Ali & Vijayan, 2020).

In terms of hydrophobic contacts, the N501Y mutation introduced an interaction with Tyr41 of ACE2 that is not present in the wild type (Fig. 3B). Other hydrophobic associations featured in the Omicron interface do not involve any mutated residues. These are similar to interactions observed in the wild type. For example, hydrophobic contacts at the N-terminus of ACE2 were conserved in both wild type and Omicron, such as S-RBD Phe486’s engagement with Leu79, Met82, and Tyr83 of ACE2 and S-RBD Tyr489’s engagement with Phe28 and Tyr83 of ACE2 (Fig. 3B). In Omicron, Phe486 interacts with Phe28 and Ala80 of ACE2 and Tyr489 interacts with Ala25, which replaces interactions with Thr27 in the wild type. In the wild type, Ala475 and Tyr473 interact with Thr27, whereas this interaction is absent in Omicron. Several recent reports have assessed the impact of variations N440K, S477N, T478K, and E484A that make no appreciable contacts in either the wild type or Omicron.

The N440K mutation has been linked to antibody resistance to mAbs (Weisblum et al., 2020), as the proximity of the N440 residue to the mAbs’ structural epitope may result in binding disruption and antibody resistance. Hence, this mutation could be associated with immune evasion (Rani et al., 2021). It has also been reported to have a higher affinity for the human ACE2 receptor and the ability to increase viral load in a relatively short period. As a result, this could be a factor in increased viral transmission (Tandel et al., 2021). Likewise, the S477N mutation was reported to improve the binding affinity of S-RBD for ACE2 and could mitigate the activity of some mAbs and convalescent plasma (Singh et al., 2021).

Concerning the T478K mutation, K478 was observed to improve the stability of the RBD–ACE2 complex. The amino acid change from a polar but uncharged threonine to a basic and charged lysine increases S-RBD’s electrostatic potential to a surface with a higher positive charge in a region that comes into contact with ACE2. Furthermore, the larger side chain of K478 may increase steric hindrance, potentially affecting the S-ACE2 interaction even more (Tandel et al., 2021). Similar to the E484K mutation’s ability to change the shape and charge of the recognition site of class two antibodies and thereby lowering antibody potency, the E484A variation has been reported to affect class one and three antibodies. The E484A mutation was reported to confer over 100-fold resistance toward the C144 mAb (Lennerstrand & Palanisamy, 2021). The S Glu484 was noted to form intermittent interactions with Lys31 of ACE2 in wild type simulations (Ali & Vijayan, 2020). This interaction was insignificant overall, with an average contact time of 30.5% across the three simulations; it was completely absent in the Omicron complex.

Unlike the Omicron variant, the Delta variant (B.1.617) features an E484Q mutation that favors improved ACE2 affinity. The L452R substitution introduced in the Delta strain creates a charged patch near the binding surfaces, allowing for increased electrostatic attraction between the S protein and ACE2. Jointly, these two mutations confer the Delta variant a higher binding affinity when compared to the wild type (Zhang et al., 2021).

Intermolecular hydrogen bonds in the Omicron complex (Fig. S5) were also compared to the wild type. Notably, the wild type complex exhibited a higher number of intermolecular hydrogen bonds (mean ± SD: 10.9 ± 2.0, 12.2 ± 1.9, 11.6 ± 1.8; Ali & Vijayan, 2020) when compared to the Omicron complex (7.6 ± 2.1, 9.0 ± 1.6, 9.7 ± 1.7). The free energy of binding (∆G_bind_) of Omicron S-RBD to ACE2 was computed using the MM-GBSA approach using frames extracted from the triplicate simulations. The results suggest that the Omicron variant possesses comparable binding affinity to ACE2 (−109.1 ± 30.6, −151.0 ± 18.9, −142.2 ± 17.8 kcal/mol) when compared to the wild type complex (−118.2 ± 13.5, −137.9 ± 11.0, −140.0 ± 10.6 kcal/mol; Ali & Vijayan, 2020) and B.1.617 variant (−147.55 ± 16.22, −132.83 ± 18.71, and −127.80 ± 21.30 kcal/mol; Antony & Vijayan, 2021). The above analysis suggests that wild type, B.1.617, and Omicron variants show similar binding strength to ACE2 (Table 1). This is in agreement with the data presented by Han et al. (2022).

**Table 1:** Comparison of the free energy of binding (∆G_bind_). Comparison of the free energy of binding (∆G_bind_) of Omicron, wild type, and B.1.617 variants obtained from molecular dynamics simulations.

Simulation run	MM-GBSA binding free energy (kcal/mol)		
	Omicron	Wild type (Ali & Vijayan, 2020)	B.1.617 (Antony & Vijayan, 2021)
Run 1	−109.1 ± 30.6	−118.2 ± 13.5	−147.6 ± 16.2
Run 2	−151.0 ± 18.9	−137.9 ± 11.0	−132.8 ± 18.7
Run 3	−142.2 ± 17.8	−140.0 ± 10.6	−127.8 ± 21.3

The structural dynamics and energetics of the wild type S protein-ACE2 complex were compared to that of the Omicron (B.1.1.529) variant. The mutations in Omicron were observed to disrupt key polar interactions featured in the RBD-ACE2 complex of the wild type structure. In comparison to the wild type, Omicron had a lower intermolecular hydrogen bond count and comparable binding free energy. In comparison to the wild type, Omicron has a slightly improved hydrophobic contact. However, this largely involves only one of the mutations in Omicron.

## Conclusions

The Omicron variant harbors a large number of variations. Some of the mutations in the S protein RBD—K417N and G496S—disrupt known interactions present in the wild type SARS-CoV-2, while others—Q493R and N501Y—introduce interactions that are absent in the original structure. Despite this, interaction at a key viral hotspot and hydrophobic contacts at ACE2’s N-terminus were well preserved. There is a drastic reduction in intermolecular polar contacts in Omicron. Thus, structural dynamics and energetics indicate that the Omicron variant may not have significantly better ACE2 binding affinity. The results presented here are based on MD simulations and additional confirmatory assays may be required to fully validate the reported findings.

## Supplemental Information

10.7717/peerj.13680/supp-1 Supplemental Information 1 Supplemental Figures.

## Additional Information and Declarations

### Data Availability

The following information was supplied regarding data availability: The simulation results from the triplicates are available in the article and Supplemental Figures.
