# Supplementary material for: Dynamics of the interaction between the receptor-binding domain of SARS-CoV-2 Omicron (B.1.1.529) variant and human angiotensin-converting enzyme 2
Source: PeerJ. 2022 Jul 5;10:e13680. doi: 10.7717/peerj.13680 (PMC9266696; doi:10.7717/peerj.13680)
Supplement: Supplemental Information 1 [file peerj-10-13680-s001.pdf]

Dynamics of the interaction between the receptor-binding domain of SARS-CoV-2 Omicron (B.1.1.529) variant and human angiotensin-converting enzyme 2

Priya Antony [1], Amie Jobe [1] and Ranjit Vijayan [1, 2, 3, *]

[1] Department of Biology, College of Science, United Arab Emirates University, PO Box 15551, Al Ain, United Arab Emirates.

[2] The Big Data Analytics Center, United Arab Emirates University, PO Box 15551, Al Ain, United Arab Emirates.

[3] Zayed Center for Health Sciences, United Arab Emirates University, PO Box 17666, Al Ain, United Arab Emirates.

* Correspondence: ranjit.v@uaeu.ac.ae; Tel.: +971 3 7136302

SUPPLEMENTARY MATERIALS

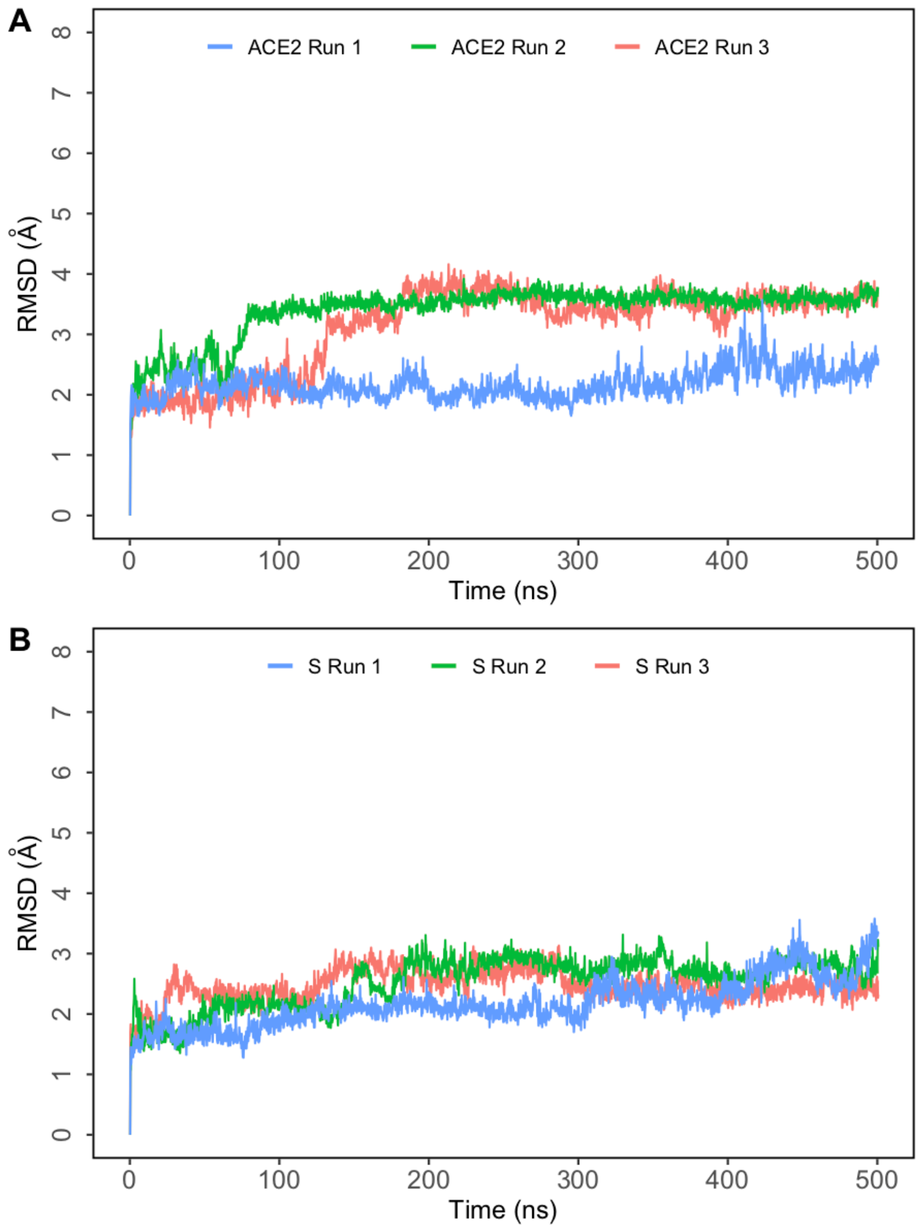

Figure S1. Root mean square deviation (RMSD) of ACE2 and Omicron spike protein. **(A)** RMSD of ACE2 protein calculated after aligning the Cα atoms of only the ACE2 chain to the initial structure. **(B)** RMSD of Omicron spike protein calculated after aligning the Cα atoms of only the S protein to the initial structure.

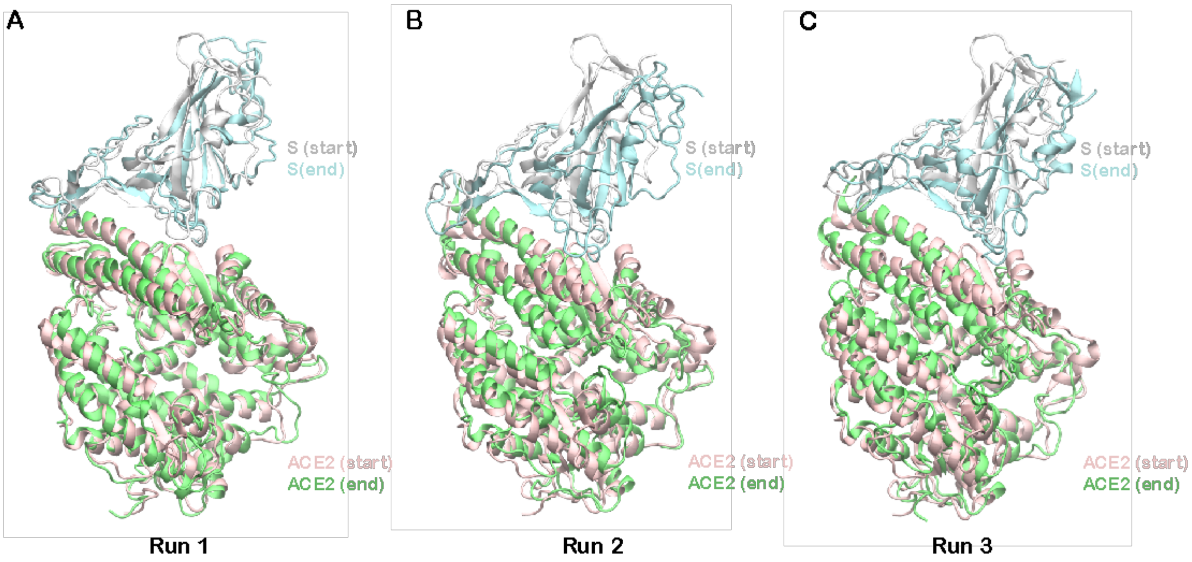

Figure S2. Comparison of the structure of the ACE2-Omicron spike protein complex from the beginning (0 ns) and end (500 ns) of the three MD simulation runs. The final structure was aligned to the initial structure in each of these images. **(A)** Run 1 **(B)** Run 2 **(C)** Run 3. In these images the protein chains are represented in cartoon representation. The start (ACE2- pink; S- gray) and end (ACE2-green; S- cyan) frames are colored differently.

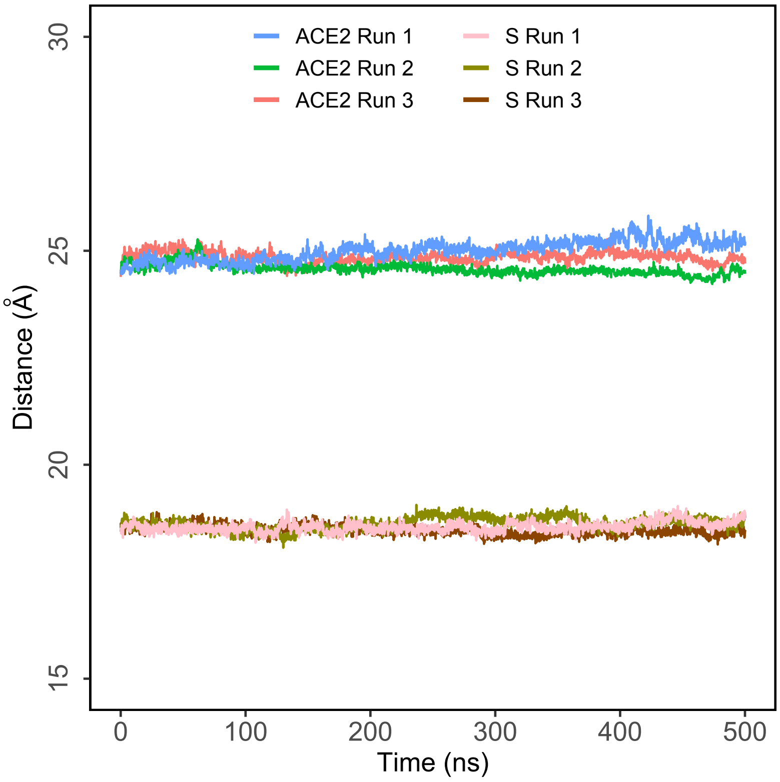

Figure S3. Radius of gyration of Omicron spike protein RBD and ACE2 from three 500 ns simulations.

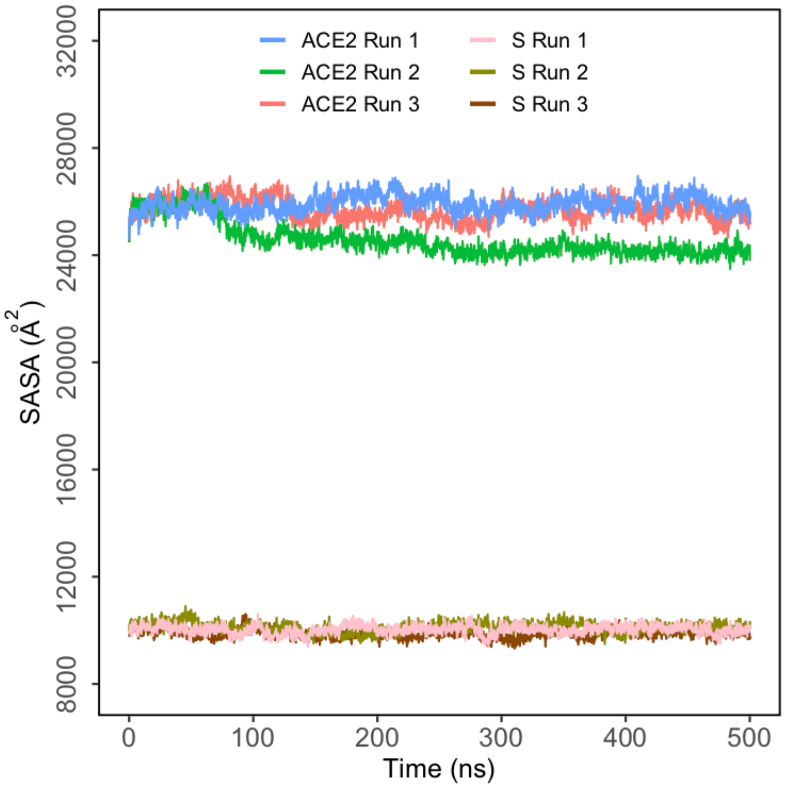

Figure S4. Solvent-accessible surface area (SASA) of ACE2 and Omicron spike protein from 500 ns simulations.

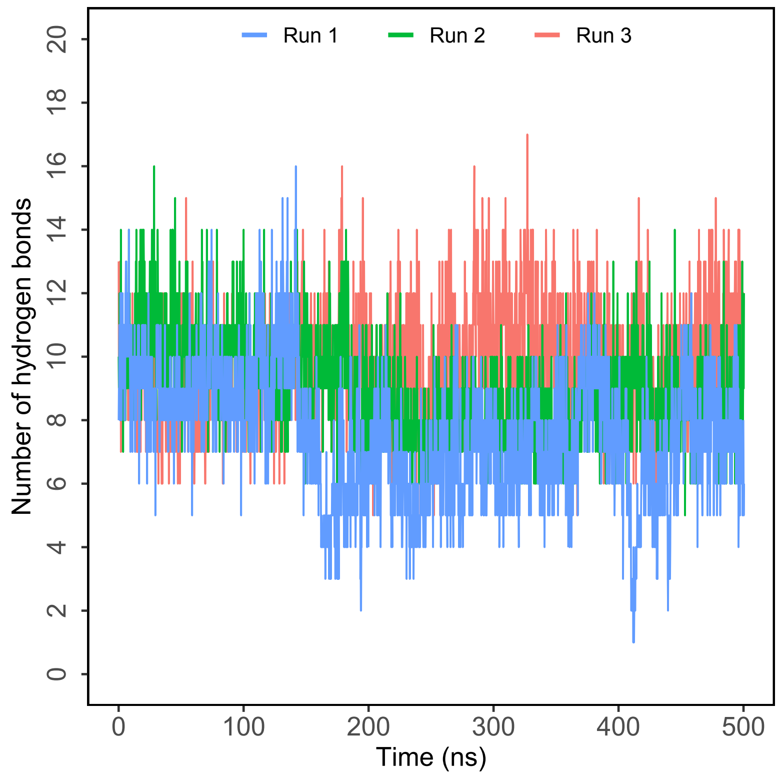

Figure S5. Intermolecular hydrogen bonds between Omicron spike protein and ACE2 from three 500 ns simulations.
